# The Intensity and Pattern of Syndecan-1 (CD138) Expression in Normal and Diseased Livers

**DOI:** 10.7759/cureus.46718

**Published:** 2023-10-09

**Authors:** Muhammad Atique, Rabia Javed, Iqtadar Seerat, Usman Atique, Tayyaba Bhatti

**Affiliations:** 1 Histopathology, Pakistan Kidney and Liver Institute and Research Center, Lahore, PAK; 2 Pediatric Gastroenterology and Hepatology, Pakistan Kidney and Liver Institute and Research Center, Lahore, PAK; 3 Pathology, Pakistan Kidney and Liver Institute and Research Center, Lahore, PAK

**Keywords:** pattern, intensity, expression, liver, syndecan

## Abstract

Introduction

Heparan sulfate proteoglycans (HSPGs) belong to the syndecan family, and syndecan-1 (CD138) is a heparan sulfate proteoglycan. Syndecan-1 has a potential role in cell-matrix and cell-cell communications as they are present in cell epithelium. Its expression is different in an extensive range of benign, inflammatory, and neoplastic diseases. In routine histopathology, it is used as a marker for plasma cells. However, it is expressed in a large variety of normal and neoplastic epithelia including squamous epithelium and gastric glandular epithelium expressed in other tissues, i.e., the liver. In the liver, variable expression is seen in cirrhosis, hepatitis, and carcinoma. The objective of this study was to investigate the expression of this marker in normal, inflammatory, and neoplastic lesions of the liver. This in turn may help clinicians to select patients who may benefit from anti-CD138 therapy. It is currently used in the diagnosis and management of plasma cell proliferations.

Material and methods

This is a retrospective study in which we retrieved 53 formalin-fixed paraffin-embedded (FFPE) liver specimen blocks and selected one block from each case by reviewing the hematoxylin and eosin (H&E) slides of each case. Syndecan-1 (CD138), pancytokeratin, and CD68 expression were analyzed immunohistochemically (IHC) to evaluate the percentage and intensity of CD138 expression in various hepatic entities and identify those entities where syndecan-1 can be consistently used to make a definitive diagnosis.

Results

The expression of pancytokeratin and CD68 was analyzed in hepatocytes and Kupffer cells, respectively. For syndecan-1 (CD138), 15.4% of cases showed basolateral membranous positivity, 44.6% of cases showed complete membranous positivity, and 40% of cases showed no positivity in hepatocytes. Cytokeratin (CK) was positive as expected in hepatocytes, and CD68 was expressed in Kupffer cells.

Conclusion

CD138 does not appear to be a reliable surrogate marker for liver disease. However, it may be included with other ancillary markers as a predictor of the stage of chronic liver disease and metastatic potential. The response to anti-CD138 therapy needs to be further studied.

## Introduction

The mammalian syndecan family consists of four members, each encoded by a distinct gene. All cell types with the possible exception of anucleate erythrocytes express at least one member of the syndecan family. Syndecan-1 is a transmembrane heparan sulfate proteoglycan (HSPG). Syndecan-1 (CD138) is mainly expressed in plasma cells and epithelial cells of human tissue. It is involved in molecular pathways that are related to cell proliferation, apoptosis, angiogenesis, and tumor invasion, so its immunohistochemical (IHC) expression is altered in a wide variety of normal and diseased tissue. Syndecan-1 (CD138) immunohistochemical (IHC) expression is correlated with the patient’s clinicopathological factors and prognosis [1].

In previous studies, in a few cancers, such as head/neck, lung, colorectal, gastric, and renal cancers, CD138 expression was found to be reduced, while multiple cancers including breast, gallbladder, pancreatic, ovarian, endometrial, urinary, bladder, and prostate cancer showed its overexpression. CD138 expression is linked to unfavorable tumor phenotype and poor patient prognosis and also to the methodology being used [2].

Syndecan-1 chains expedite intercellular signaling by binding numerous molecules; providing for scaffolding of vital connections in liver fibrosis are the ones with transforming growth factor-β1 (TGFβ1), hepatocyte growth factor (HGF), and basic fibroblast growth factor (bFGF). Cirrhotic patients can be evaluated using syndecan-1 [3]. Syndecan-1 shedding inhibits liver fibrogenesis. These shedded ectodomains of syndecan-1 bind and boost TGFβ1 clearance and facilitate fibrogenesis. In contrast, the protective effect of syndecan-1 lasts for 2-4 months, and then, immunochemistry (IHC) senses small levels of heparan sulfate (HS) chains, suggestive of an exhausted synthesis of syndecan-1 [4].

In addition, serum levels of syndecan-1 are raised in hepatocellular carcinoma (HCC) patients compared with cirrhotic patients [5]. A study by Baghy et al. revealed only a small increase in syndecan-1 expression in cancer without cirrhosis, compared with cancer with cirrhosis, which, in contrast, presented a considerably higher expression of syndecan-1 [6].

Syndecan-1 levels are increased in HCC both with and without previous cirrhosis; its expression is significantly greater in the former, with high levels also detected in the peritumoral cirrhotic areas. Concerning the correlation between syndecan-1 levels and the tumor stage, the correlation was not statistically significant [7].

Viral internalization takes place through direct interaction between syndecan-1 and CD81 at the basolateral level of the hepatocyte membrane. This finding was reinforced by the observation that the blocking of syndecan-1 inhibited hepatitis C virus (HCV) infection, while the knocking down of both syndecan-1 and CD81 exerted an even more powerful inhibitory action upon viral internalization [8].

Syndecan-1 (CD138) expression in different cancers is of upcoming clinical interest as precise drugs targeting CD138 are now being evaluated in clinical trials, particularly in plasma cell neoplasms. If anti-CD138 therapies should prove successful, other CD138-positive cancer types might as well benefit from such treatments [2].

In this present study, we have investigated the percentage and intensity of syndecan-1 expression in normal liver and various hepatic pathologies.

We hope that our study enhances the already existing knowledge about the utility of syndecan-1 as a surrogate marker for hepatocytes as well as its diagnostic and therapeutic utility in various hepatic lesions.

## Materials and methods

Hematoxylin and eosin (H&E) slide analysis and preparation of IHC slides

After approval by the institutional review board (number: PKLI-IRB/AP/022), H&E-stained slides of liver specimens from August 2019 to March 2020 were retrieved from the archives of the Department of Histopathology at Pakistan Kidney and Liver Institute and Research Center (PKLI&RC), including normal liver, non-tumorous and tumorous parts of a liver, and other lesional areas, including cirrhotic liver.

Appropriate blocks including the maximum representative tissue were selected for performing immunohistochemistry (IHC). Syndecan-1 immunohistochemistry was performed on the selected formalin-fixed paraffin-embedded (FFPE) blocks.

Three sections from each selected block were taken on positively charged slides and rehydrated by following dewaxing and treatment with graded alcohol (100%, 90%, 80%, and 70%).

Immunohistochemical (IHC) procedure

Heat-Induced Epitope Retrieval (HIER) Procedure

Antigen retrieval was performed in a water bath using Bond Epitope Retrieval Solution 2 (Leica Biosystems, Wetzlar, Germany) at 98°C for 30 minutes. The immunohistochemistry (IHC) procedure was performed at room temperature using Novolink Max Polymer DS (Leica Biosystems) (1,250) kit.

Immunohistochemistry (IHC) Procedural Steps

After retrieval, slides were placed in distilled water and allowed to cool down. Then, endogenous peroxidase was blocked using RE7175 peroxidase block for five minutes and then washed twice in phosphate buffer saline (PBS) for five minutes each. To prevent any non-specific binding, sections are treated with RE7158 protein block for five minutes. For washing, slides were placed in PBS for five minutes (two changes). For CD138 slides, pre-diluted, ready-to-use mouse anti-human monoclonal CD138 (MI 15) antibody (Leica Biosystems) was applied on both positive control and test tissues for 30 minutes at room temperature. The slides were washed twice in PBS for five minutes each. Afterward, all slides were incubated with RE159 post-primary reagents for 30 minutes at room temperature, followed by washing twice in PBS for five minutes. All slides were then incubated with RE161 Novolink TM Polymer at room temperature for 30 minutes. The immunohistochemical reaction was identified by RE7163 Novolink DAB Substrate Buffer, according to the company guidelines. The slides were washed with distilled water and then counterstained with hematoxylin. After tap water washing, the slides were dehydrated in three graded alcohol, cleared in xylene, and ultimately mounted with dibutyl phthalate polystyrene xylene (DPX).

## Results

Syndecan is the major proteoglycan of the liver and plays a critical role in the clearance of lipoproteins from the circulation, acting as part of the protein complex of the very low-density lipoprotein (VLDL) receptor [2].

**Figure 1:**
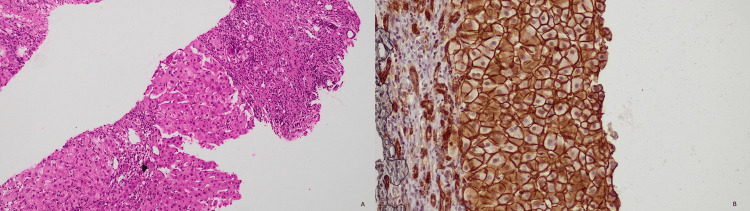
(A) Biliary pattern cirrhosis 20× magnification and (B) complete membranous staining with CD138

A total of 65 cases were included in this study, of which 30 were male and 35 were female patients, with ages ranging from four to 73 years and with an average age of 40.67±18.1 years. There were 33 cases with inflammatory conditions, 13 with cirrhosis, eight with benign neoplasms, and 11 with malignant neoplasms. Table 1 shows the diagnoses in each category. The results of CD138 staining (Figure 1) were as follows: 30 with membranous positivity, seven with basolateral positivity, and 28 with negative staining. Table 2 gives the distribution of patterns of staining among the main diagnoses.

**Table 1:** Lesions included for testing with syndecan-1

Inflammatory	Cirrhosis	Neoplastic (benign)	Neoplastic (malignant)
Primary biliary cholangitis	Liver cirrhosis	Hepatocellular adenoma	Cholangiocarcinoma
Granulomatous inflammation		Simple biliary cyst	
Hydatid cyst		Focal nodular hyperplasia	Hepatocellular carcinoma
Chronic viral hepatitis	Biliary cirrhosis	Bile duct hamartoma	
Sclerosing cholangitis			Metastatic adenocarcinoma
Glycogen storage disease			

**Table 2:** Syndecan-1 expression in various liver lesions (N=65)

Inflammatory (n=33)		Cirrhosis (n=13)		Benign neoplastic conditions (n=8)		Malignant neoplastic conditions (n=11)		p-value
Strong membranous	15 (45.5%)	Strong membranous	8 (61.5%)	Strong membranous	3 (37.5%)	Strong membranous	4 (36.4%)	0.591
Basolateral	5 (15.2%)	Basolateral	0 (0%)	Basolateral	0 (0%)	Basolateral	2 (18.2%)	
Negative	13 (39.4%)	Negative	5 (38.5%)	Negative	5 (62.5%)	Negative	5 (45.5%)	

## Discussion

Over the years, various immunohistochemical markers have been studied with respect to liver disease, and some surrogate markers have been identified. These include HepPar1, glutamine synthetase, arginase 1, and glypican 3. In our study, we aimed to assess the expression of syndecan-1 (CD138) in various liver diseases. Syndecans form a family of cell surface glycoproteins that can interact with various effector molecules such as extracellular matrix and growth factors [9].

While our study showed that most cases of primary hepatocellular carcinomas were positive for CD138, one case of metastatic hepatocellular carcinoma was negative. This was in keeping with the study by Matsumoto et al. [10]. Harada et al. also found reduced expression of CD138 in poorly differentiated and metastatic cholangiocarcinoma, similar to our study [11]. A possible hypothesis for this is that syndecan-1 expression is associated with retention of epithelial morphology and inhibition of invasiveness in vitro. Loss of expression would result in increased invasiveness and risk of metastasis [9]. These prognostic features have also been described in bladder cancer where increased CD138 expression in stromal cells and loss of expression in tumor cells were associated with the progression of bladder cancer; however, this is yet to be confirmed with broader studies with comparisons and pairwise analysis through expression profiling [12].

Our study also found increased and complete CD138 expression in advanced stages of chronic liver disease such as cirrhosis. This has been previously discussed by Charchanti et al. in their study on advanced chronic liver disease where they found that CD138 expression was positively correlated with advanced stages of chronic liver disease [13]. Similar findings were reported by Regős et al. [7].

In our research, we found that all hepatocellular carcinomas show increased expression of CD138 in hepatocytes and peritumoral inflammatory cells. This makes it a potential target for therapy; in fact, cabozantinib and nivolumab (anti-CD138 agents) were found to convert advanced disease to potentially resectable disease. Considering this, CD138 may be included in routine prognostic evaluation of hepatocellular carcinomas [14]. There was no statistically significant difference between the groups studied with regard to staining pattern, so it is unlikely to help in differentiating between these groups.

From this study, most of the lesions of liver inflammation, cirrhosis, and hepatocellular carcinoma show either complete or basolateral membranous positivity. However, many of the liver lesions are negative in about 40% of the cases, so this IHC antibody may not be used as a surrogate marker for hepatocellular lesions.

This study was limited by the small number of cases, and a larger study with a more extensive disease range should be done to study the expression of syndecan-1 in liver diseases.

## Conclusions

Syndecan-1 is not a reliable marker for differentiating the etiology of liver disease. However, a more extensive yet focused study can be performed to assess the pattern of staining in various diseases. Considering the positivity in liver tissue, it should be used with caution for plasma cell detection. Considering the variable positivity in hepatocellular carcinomas, the option of anti-CD138 therapy can be studied through clinical trials correlating with the pattern and extent of expression. It may, in the future, be a treatment option for hepatocellular carcinomas resistant to conventional chemotherapeutic agents.
